# P-1894. Unifying the Frontline: System-Wide Antimicrobial Pharmacist Education Implementation

**DOI:** 10.1093/ofid/ofaf695.2063

**Published:** 2026-01-11

**Authors:** Erin Weslander, Rishita Shah, Emvica Cash, Jason Park, Radhika S Polisetty, Sheila K Wang, Michael Diakoumis, Michael Postelnick, Christie M Bertram, Jaime Borkowski, Stephanie Chang, Michael Dickens, William Justin Moore, Aaron Oliver, Sarah Hale Sutton

**Affiliations:** Northwestern Memorial Hospital, Chicago, IL; Northwestern Lake Forest Hospital, Lake Forest, IL, Illinois; Northwestern Medicine, Chicago, Illinois; Northwestern Medicine, Chicago, Illinois; Midwestern University College of Pharmacy/ Northwestern Medicine Central DuPage Hospital, Winfield, Illinois; Midwestern University College of Pharmacy/Northwestern Memorial Hosptial, Downers Grove, Illinois; Northwestern Medicine, Chicago, Illinois; Northwestern Medicine, Chicago, Illinois; Northwestern Memorial Hospital/Rosalind Franklin University of Medicine and Science, Chicago, Illinois; NM Delnor Hospital, Geneva, Illinois; Northwestern Medicine - Huntley Hospital, Huntley, Illinois; Northwestern Medicine, Chicago, Illinois; Northwestern Medicine, Chicago, Illinois; Northwestern Medicine, Chicago, Illinois; Northwestern Memorial Hospital, Chicago, IL

## Abstract

**Background:**

Clinical pharmacists are key contributors to Antimicrobial Stewardship (AMS) program efforts. Baseline knowledge of infectious diseases among hospital pharmacists across an 11-hospital system was inconsistent due to several factors including residency training, year since educational training, site-specific onboarding procedures, and more. Based on Joint Commission Standard MM.09.01.01 EP12, hospitals must document AMS competency for staff. To address these gaps, we implemented an annual pharmacist competency “The Antimicrobial Resistance Fighter Curriculum” with the first round consisting of 3 modules.

**Image 1:**
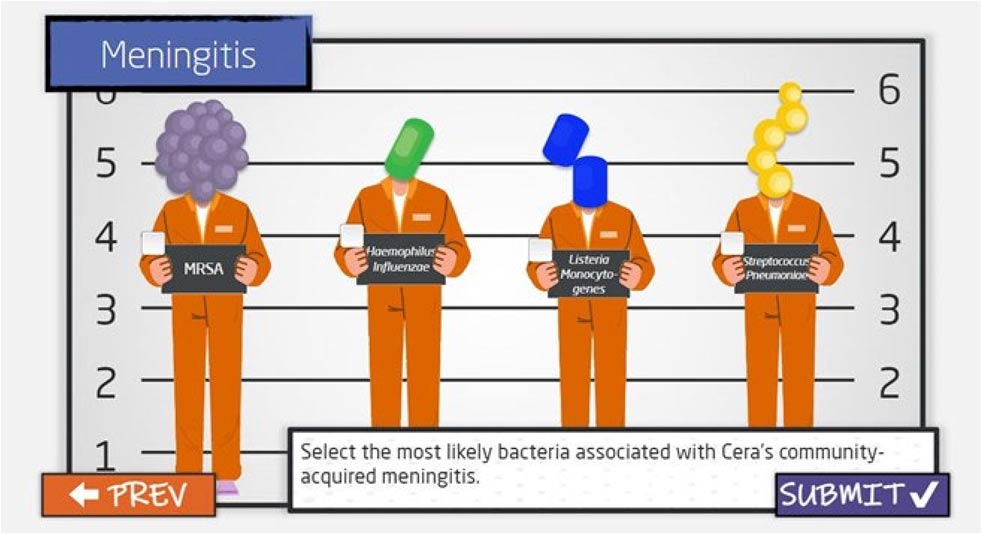
Example of Knowledge Check Question 1

**Image 2:**
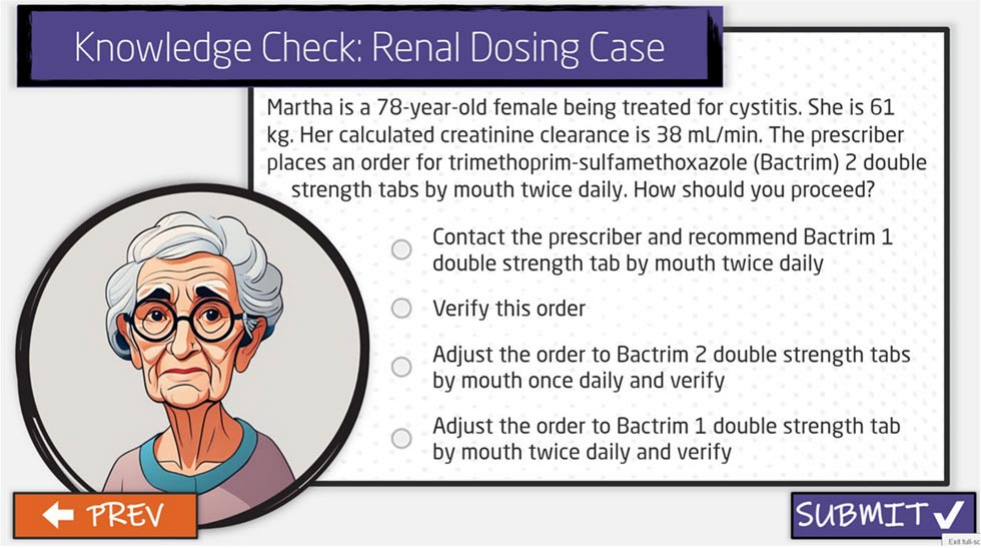
Example of Knowledge Check Question 2

**Methods:**

A multi-disciplinary working group within ADSP developed competency content for the antimicrobial stewardship concepts. Feedback from stakeholders and frontline users was incorporated prior to the launch to ensure objectives were being met. Knowledge retention was reinforced through built-in knowledge checks and creative graphics (Images 1,2).

Baseline metrics were also assessed on a scale of 1-5 (1= no knowledge, 5= confident using knowledge daily) before and after implementation. New hire education began May 2024, and system wide education was assigned on August 27^th^, 2024, to any remaining pharmacists. We also tracked the competency of staff by a mandatory 100% quiz pass rate. This curriculum is also applied during the on-boarding period for new pharmacists.

**Table 1:**
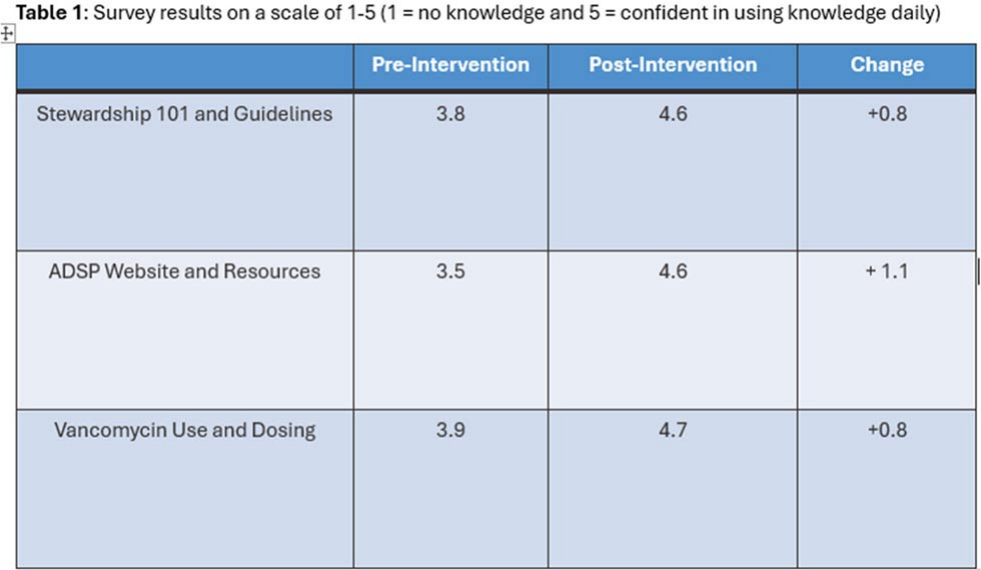
Survey results on a scale of 1-5 (1 = no knowledge and 5 = confident in using knowledge daily)

**Image 3:**
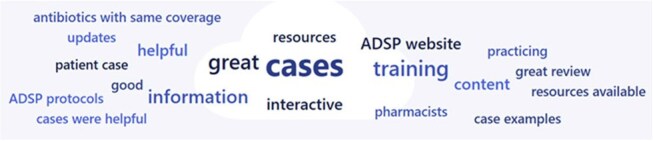
Word cloud of comments from pharmacist survey question "What parts of this training did you find most effective? Do you have any suggestions on how to make this content better?"

**Results:**

415 pharmacists across the health system completed the curriculum. Confidence in using AMS topics in daily practice increased from an average of 3.8 to 4.6 out of 5 for Module 1:Antimicrobial Stewardship 101 and Guidelines, 3.5 to 4.6 for Module 2:ADSP Website and Resources, 3.9 to 4.7 for Module 3:Vancomycin Use and Dosing (Table 1). We had 93 responses to a second survey 9 months after the launch of the competency to assess if end users still felt that our competency was useful for daily patient care activities and found 1% did not make changes, 5% were planning to make changes, and 78% made changes to their clinical practice due to this education.

**Conclusion:**

This type of competency-based intervention demonstrates that antimicrobial stewardship knowledge can improve across a healthcare system. The content was well-received, and many pharmacists appreciated the creative learning style (Image 3).

**Disclosures:**

All Authors: No reported disclosures

